# Diet-Induced Obesity Induces Transcriptomic Changes in Neuroimmunometabolic-Related Genes in the Striatum and Olfactory Bulb

**DOI:** 10.3390/ijms25179330

**Published:** 2024-08-28

**Authors:** Rosario B. Jaime-Lara, Claudia Colina-Prisco, Marcel De Jesus Vega, Sarah Williams, Ted Usdin, Bridget Matikainen-Ankney, Alayna Kinkead, Brianna Brooks, Yupeng Wang, Alexis T. Franks, Alexxai Kravitz, Paule V. Joseph

**Affiliations:** 1 National Institute of Alcohol Abuse and Alcoholism, Bethesda, MD 20892, USA; rjaime-lara@mednet.ucla.edu (R.B.J.-L.);; 2 National Institute of Nursing Research, Bethesda, MD 20892, USA; 3 School of Nursing, University of California, Los Angeles, CA 90095, USA; 4 National Institute of Mental Health, Bethesda, MD 20892, USAusdint@mail.nih.gov (T.U.); 5 Department of Psychology, Rutgers University, Piscataway, NJ 08854, USA; 6 Department of Psychiatry, Washington University in St. Louis, St. Louis, MO 63110, USA; 7 National Smell and Taste Center, National Institute on Deafness and Other Communication Disorders, Bethesda, MD 20892, USA

**Keywords:** diet, obesity, transcriptome, striatum, olfactory bulb

## Abstract

The incidence of obesity has markedly increased globally over the last several decades and is believed to be associated with the easier availability of energy-dense foods, including high-fat foods. The reinforcing hedonic properties of high-fat foods, including olfactory cues, activate reward centers in the brain, motivating eating behavior. Thus, there is a growing interest in the understanding of the genetic changes that occur in the brain that are associated with obesity and eating behavior. This growing interest has paralleled advances in genomic methods that enable transcriptomic-wide analyses. Here, we examined the transcriptomic-level differences in the olfactory bulb and striatum, regions of the brain associated with olfaction and hedonic food-seeking, respectively, in high-fat-diet (HFD)-fed obese mice. To isolate the dietary effects from obesity, we also examined transcriptomic changes in normal-chow-fed and limited-HFD-fed groups, with the latter being pair-fed with an HFD isocaloric to the consumption of the normal-chow-fed mice. Using RNA sequencing, we identified 274 differentially expressed genes (DEGs) in the striatum and 11 in the olfactory bulb of ad libitum HFD-fed mice compared to the chow-fed group, and thirty-eight DEGs in the striatum between the ad libitum HFD and limited-HFD-fed groups. The DEGs in both tissues were associated with inflammation and immune-related pathways, including oxidative stress and immune function, and with mitochondrial dysfunction and reward pathways in the striatum. These results shed light on potential obesity-associated genes in these regions of the brain.

## 1. Introduction

Obesity has a pronounced impact on morbidity and mortality, as it contributes to the global incidence of chronic diseases, including cardiovascular disease, type 2 diabetes and cancer [1]. The rise in the global incidence of obesity over the last several decades is attributed to an increased availability of highly palatable and energy-dense foods, including high-fat foods [2,3]. The hedonic value of energy-dense foods promotes preferential consumption, leading to increased caloric intake and obesity [4].

One potential mechanism by which palatable foods can promote greater eating behavior is through the reinforcing properties of chemical stimuli, which vertebrates can sense through olfactory receptor neurons [5]. In the olfactory bulb, olfactory receptor neurons constitute the first centrally mediated steps in processing olfactory signals [6]. In turn, these olfactory signals play a dominant role in flavor perception [7]. The striatum is a vital reward center of the brain, and studies have demonstrated that it receives indirect olfactory input from the amygdala [5]. These projections could constitute a neural basis by which pleasant olfactory stimuli (e.g., from food) are reinforced [5], thereby motivating eating/ingestive behaviors. Thus, the olfactory bulb and striatum potentially support energy homeostasis regulation and obesogenic factors by integrating the chemical senses and reward pathways.

Advances in genomic methods have allowed for an exploration of the molecular changes contributing to obesity. Although genetic studies of obesity have identified multiple mutations and loci associated with obesity (e.g., the ob/ob mouse, which has mutations in the gene encoding for leptin), exploratory studies of genome-wide changes can help to leverage system-level gene expression changes. System-based approaches may offer important insights into the biological pathways contributing to obesity. Other system-based approaches, such as weighted gene correlation network analysis (WGCNA), can allow for the further investigation of the genomic mechanisms underlying obesity by enabling the examination of groups of co-expressed genes associated with variables of interest (e.g., body mass index).

Furthermore, there is an underutilization of systems-based approaches in genomic studies of obesity in brain tissue. Few studies have examined genomic changes in brain tissue in obese animal models. Previous studies have examined some brain regions, including the mouse brain cortex [8] and the hypothalamus [9]. However, to the best of our knowledge, no transcriptomic sequencing studies have been conducted in the olfactory bulb or striatum of diet-induced obese mice. As these regions are involved in eating behavior and energy balance, studying gene expression changes in the striatum and olfactory tissue could be important for determining if there are centrally mediated transcriptomic changes induced by obesity. Therefore, here, we aimed to examine transcriptomic-level differences (e.g., differentially expressed genes (DEGs) and weighted gene co-expression networks) in the striatum and olfactory bulb of obese mice following the consumption of a high-fat diet (HFD). To disentangle the effects of obesity from the diet per se, we also examined transcriptomic changes in these brain regions following a normo-caloric, limited high-fat diet (limited HFD) and a dietary intervention (HFD-to-chow).

## 2. Results

**Figure 1:**
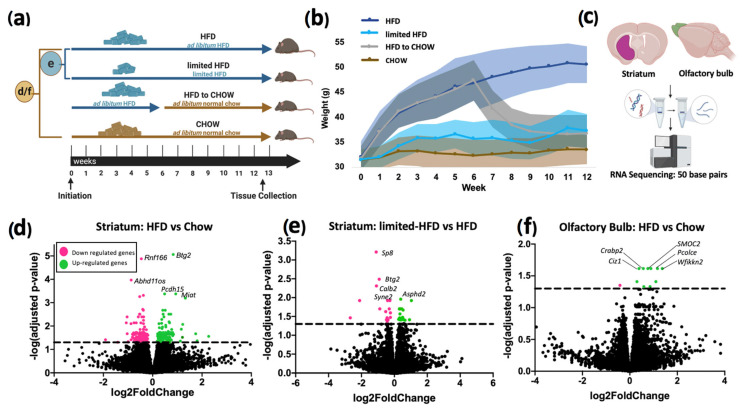
Study design and differentially expressed genes: (**a**) study design. Letters d/f, and e correspond with DE gene volcano plots below; (**b**) animal weights over time; (**c**) representation of the striatal and olfactory bulb tissue used for RNA Sequencing; (**d**) volcano plot showing 274 differentially expressed genes between the high-fat diet group and the chow group; (**e**) volcano plot showing 38 differentially expressed genes between high-fat diet group and the limited high-fat diet group and (**f**) volcano plot showing 11 differentially expressed genes between high-fat diet group and the limited high-fat diet group.

After two weeks of exposure to an ad libitum HFD, the HFD animals displayed a significantly higher body weight compared to control and limited HFD groups. The HFD-to-chow group’s body weight dropped after week six and was not significantly different to the control (chow) group by week 12 (Figure 1a,b). RNA sequencing of striatal and olfactory tissue (Figure 1c) identified 274 DEGs between the ad libitum HFD and chow groups in the striatum (Figure 1d), 38 DEGs between the ad libitum HFD and limited HFD groups (Figure 1e), and 11 DEGs in the olfactory bulb of ad libitum HFD mice compared to the chow group (Figure 1f). In general, HFD and chow groups had the highest number of DEGs and showed greater separation among the diet groups; HFD-to-chow was closer to HFD; and limited HFD was closer to chow. (Figure 1 and Supplementary Materials Figure S7).

### 2.1. DEGs in the Striatal Tissue

Of the 274 DEGs between the ad libitum HFD and the chow groups (Table 1), the top five DEGs were, as follows: B-cell translocation gene 2 (BTG2); Ring Finger Protein 166 (Rnf166); ABHD11 Antisense RNA 1 (Abhd11os); Protocadherin Related 15 (Pcdh15); and Myocardial Infarction Associated transcript (Miat). The top five DEGs were highly statistically significant after adjusting for multiple comparisons, with adjusted *p* values ranging from 8.66 × 10^−6^ to 4.22 × 10^−4^. Fold change among these 274 genes ranged from |2.68| to |0.17|, and 117 of these genes showed a small to moderate fold change of 0.5 or greater, indicating subtle but potentially biological significant differences. Btg2 was upregulated and is involved in mitochondrial depolarization, which plays a role in the mediation of mitochondrial reactive oxygen species [10] and in neurite outgrowth [11]. Rnf166 was downregulated and is involved in interferon production or autophagy and affects cellular responses, including cell growth and inflammation. Abh11os was downregulated and is involved in oncogenesis through its role in cell proliferation and decreased apoptosis via STAT3 [12]. Pcdh15 was upregulated and has been associated with lipid levels in familiar hyperlipidemia [13]. Miat was upregulated and altered expression of this locus has been associated with susceptibility to myocardial infarction via its effect on oxidative stress [14].

The GO term enrichment of these 274 DEGs identified multiple molecular functions (e.g., NADH dehydrogenase activity, oxidoreductase activity and electron transfer activity) and cellular components (mitochondrial respiratory complex 1, respiratory chain complex 1, and NADH dehydrogenase complex) (Table S1).

**Figure 2:**
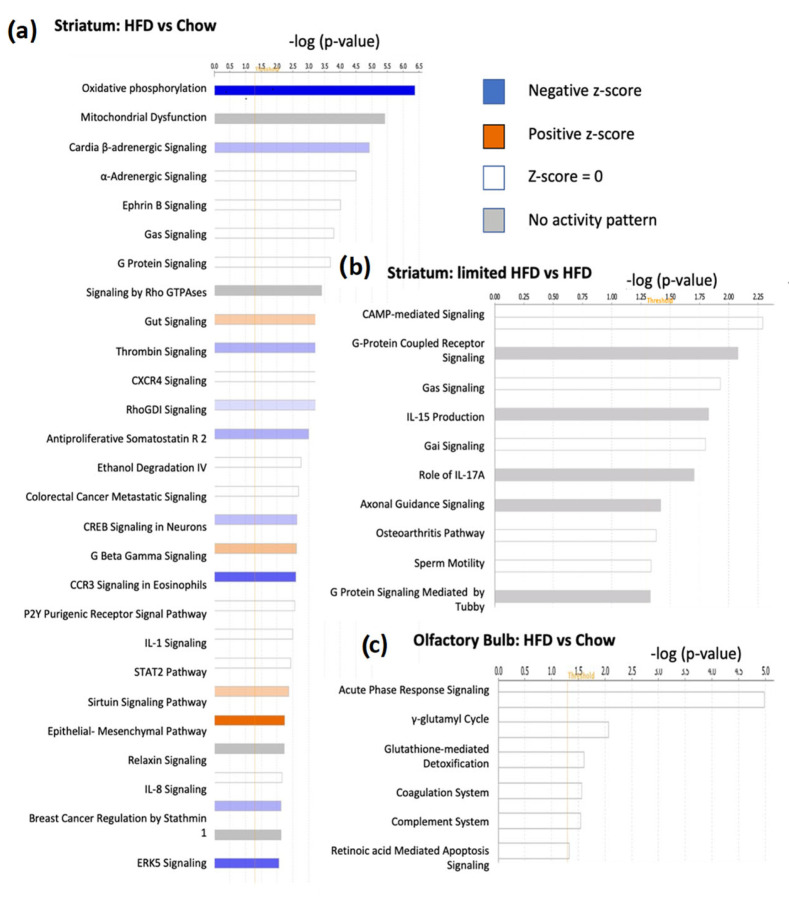
Study design and differentially expressed genes: (**a**) top 25 canonical pathways of DE genes between HFD and chow groups in the striatum; (**b**) all canonical pathways of DE genes between the limited HFD and HFD groups; and (**c**) all canonical pathways between the HFD and chow groups in the olfactory bulb. Figure was generated using IPA (QIAGEN Inc; Redwood City, USA.) https://www.quiagenbio-informatics.com/producs/ingenuity-pathways-analysis (accessed on 4 April 2024).

**Figure 3:**
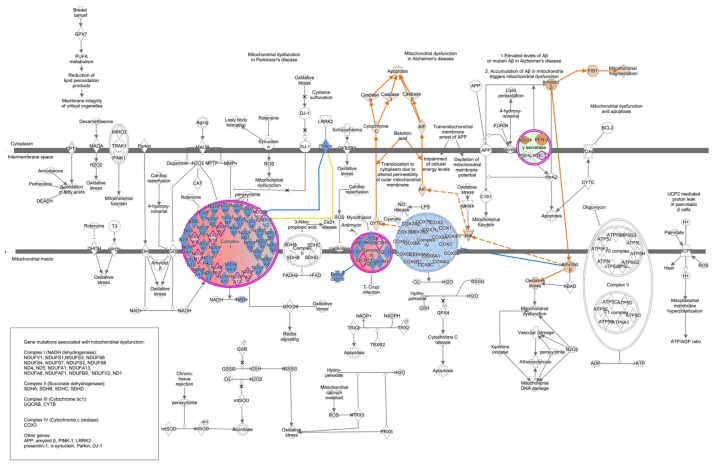
Oxidative phosphorylation and mitochondrial dysfunction canonical pathways. Genes in red represent upregulated genes. Genes in green are downregulated genes. Colored arrows show the results of the molecule activity predictor; orange lines represent pathway activation; blue lines represent inhibition; yellow lines represent conflicting data.

IPA Pathway and network analyses of the 274 DEGs identified these genes as being involved in 17 networks (Supplementary Figure S4A), 64 canonical pathways and 63 diseases and functions. Gene networks were generated and scored based on the relevancy of genes with known biological functions, with the highest possible score of 50. The top three gene networks of these DEGs were as follows: (1) cell signaling, post-translational modification, protein synthesis (score of 42); (2) cell morphology, cell-to-cell signaling and interaction, cellular assembly and organization (score of 37); and (3) cancer, organismal injury and abnormalities, and inflammatory response (score of 35). The top canonical pathways included oxidative phosphorylation, mitochondrial dysfunction and cardiac ß-adrenergic signaling (Figure 2a). The molecule activity predictor analyses of the top two canonical pathways predicted that the DEGs in these canonical pathways increase the activity of pathways involved in apoptosis, oxidative stress, and mitochondrial fragmentation (Figure 3). The top three diseases and functions associated with the 274 DEGs were cancer, gastrointestinal disease, and organism injury and abnormalities (Supplementary Figure S5).

There were also 38 DEGs between the ad libitum HFD and the limited HFD groups (Table 1). The top five DEGs were highly statistically significant after adjusting for multiple comparisons, with adjusted *p* values ranging from (6.12 × 10^−4^ to 0.01). Fold change among these 38 genes ranged from |0.24| to |2.68|, and 14 of these genes showed a small to moderate fold change of 0.5 or greater, indicating small but biological significant differences. These 38 genes included SP8 Transcription Factor (Sp8) and BTG Anti-Proliferation Factor 2 (Btg2), Calbindin 2 (Calb2), Aspartate Beta-Hydroxylase Domain Containing 2 (Asphd2) and Spectrin Repeat Containing Nuclear Envelope Protein 2 (Syne 2). Sp8 was downregulated and is a transcription factor that is important for the generation of dopamine 2 medium spiny neurons (D2 MSNs) [15]. Additionally, Btg2 was also differentially expressed between the ad libitum HFD and limited HFD groups. Calb2, which was downregulated, is a calcium-binding protein involved in neuronal excitation, including long-term potentiation [16]. Asphd2, which was upregulated, enhances cell proliferation, migration, and invasion [17,18]. It also reportedly affects mitochondrial function by disrupting mitochondrial DNA stability [19]. Syne2 was downregulated and is integral to nuclear ERK1/2 signaling, cell cycle progression and neuronal migration [20,21].

**Figure 4:**
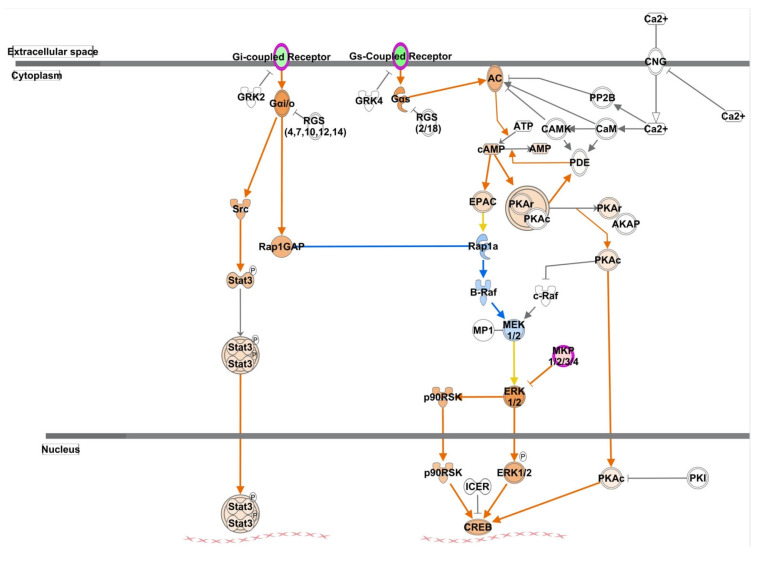
cAMP-mediated signaling canonical pathway. Genes in red represent upregulated genes. Genes in green are upregulated genes. Colored arrows show the results of the molecule activity predictor; orange lines represent pathway activation; blue lines represent inhibition; yellow lines represent conflicting data.

Go enrichment identified that the top GO terms included positive regulation of synaptic transmission (GABAergic), low-density lipoprotein receptor particle metabolic process and regulation of low-density lipoprotein particle receptor catabolic process. IPA analyses found these 38 DEGs are also involved in 4 networks (Supplementary Figure S4B), 10 canonical pathways (Figure 2b) and 69 diseases and functions (Supplementary Figure S5B). The top networks included, as follows: (1) cellular movement, amino acid metabolism, and molecular transport (score of 36); and (2) cancer, organismal injury and abnormalities, and renal and urological disease (score of 33). The top networks included Adamts1, Ddr2, Hrh3, Kdm4b, Slc7a11, Adora2a, Dusp1, Pcsk9, S100a8, Syne2, Erbb4, Scgn, Wls, Btg2 and Sema3f. The top three canonical pathways were, as follows: cAMP-mediated signaling (Figure 4); G-protein coupled receptor signaling; and gαs signaling. The DEGs included those involved in acid metabolism, molecular transport, and small molecule biochemistry.

We identified two DEGs between the HFD-to-chow and the limited HFD groups; namely, Histocompatibility Complex, Class II, Cd74, and Eukaryotic translation initiation factor 3, EiF3j1. Cd74 plays a critical role in MHC Class II antigen processing and is a plasma membrane receptor for the macrophage migration inhibitory factor (MIF) [22]. EiF3j1 participates in the initiation of translation by mRNAs involved in cell proliferation, including differentiation and apoptosis [23]. We did not find any DEGs between the ad libitum HFD and the HFD-to-chow groups, between the chow and HFD-to-chow groups nor the chow and limited HFD groups.

### 2.2. DEGs in the Olfactory Tissue

The transcriptomic analyses showed that diet was associated with DEGs in the olfactory bulb tissue. We identified eleven DEGs between the ad libitum HFD and chow groups (Table 2). No DEGs were identified between the ad libitum HFD and HFD-to-chow groups, the chow and HFD-to-chow groups, nor in the chow and limited HFD groups. The top five DEGs had adjusted *p* values ranging from (0.02 to 0.04). Fold change among these 11 genes ranged from |0.30| to |1.37|, and these genes showed a small to moderate fold change of 0.5 or greater, indicating small but biologically significant differences. These top five DEGs were, as follows: SPARC-related modular calcium binding 2 (Smoc2); CDKN1A interacting zinc finger protein 1 (Ciz1); procollagen C-endopeptidase enhancer (Pcolce); WAP, follistatin/kazal, immunoglobulin, kunitz and netrin domain containing 2 (Wfikkn2); and cellular retinoic-acid-binding protein 2 (Crabp2). Wfikkn2 is a protease inhibitor that is important for regulating serine and myostatin activity [24]. A recent study identified six proteins (LEPR, IGFBP1, WFIKKN2, AGER, DPT, and CTSA), including WFIKKN2, which may have a causal role in the development of obesity [24]. Pcolce (also known as PCEP) binds procollagen and improves procollagen C-proteinase function, affecting resting and post-exercise heart rate and recovery. Its paralog gene, PCPE2, plays a vital role in regional fat deposition linking SR–BI-mediated cholesterol ester uptake to adipose cell biology [25]. Smoc2 has been reported to be involved in multiple cellular processes, including fibrosis, inflammation, cell differentiation, cell proliferation, and lipid deposition observed in metabolic syndrome [26,27]. Crabp2 is involved in immune regulation and inflammation [28].

The top GO enrichment terms associated with DEGs and molecular functions or cellular components, and the best GOs included retinoic acid-binding and cyclin binding. Network and pathway analyses of the eleven DEGs conducted using IPA identified one network and four canonical pathways. The network was cellular development, connective tissue development and function, and tissue development (score of 30), including all genes listed above and Stra6, Fn1, Ndrg1, Anpep, and Steap3. The top canonical pathways included acute phase response signaling (Supplementary Figure S3), gamma-glutamyl cycle and glutathione-mediated detoxification (Figure 2c). The IPA molecular activity predictor suggested the activation of inflammatory pathways. The DEGs were involved in 72 categories of diseases and functions, with the most significant being cancer, organismal injury and abnormalities, cell death and survival, embryonic development, and cell morphology.

There were no DEGs found in striatal tissue between the HFD-to-chow and the limited HFD groups, between the ad libitum HFD and the HFD-to-chow groups, between the chow and HFD-to-chow groups nor between the chow and limited HFD groups.

### 2.3. Weighted Gene Co-Expression Network Associated with Free Fatty Acids

**Figure 5:**
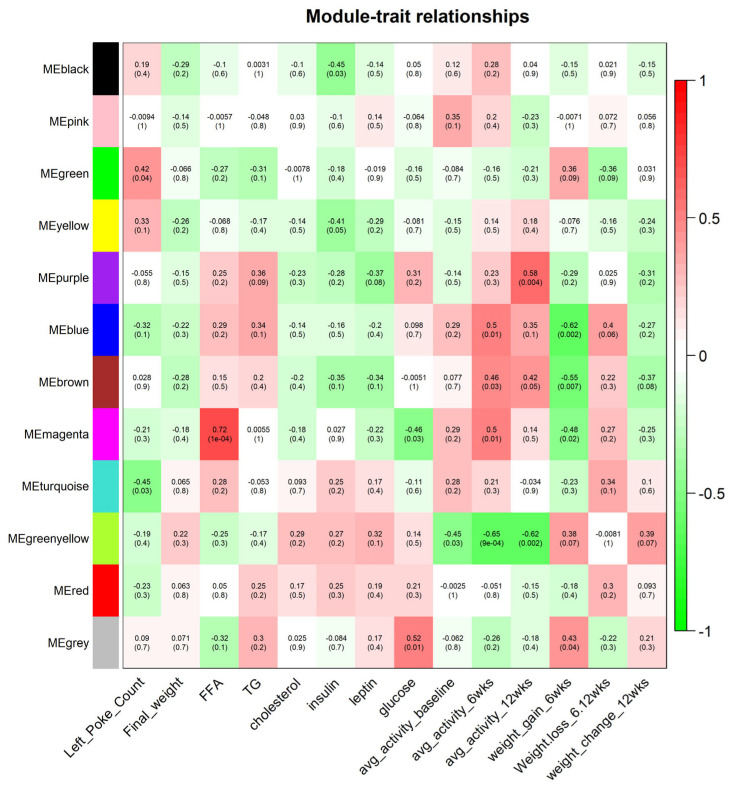
Gene co-expression networks and clinical characteristics in olfactory tissue. Colors along y axis represent gene co-expression networks. Clinical traits are listed along x axis. FFA: free fatty acids, TG: triglycerides, avg: average, wks: weeks.

One gene module, the magenta module, was found to be associated with biological variables (Figure 5). The magenta module was associated with free fatty acid levels. Top hub genes within the magenta module were early growth response protein 3 (Egr3), protein FOSB (Fosb) and nuclear receptor subfamily 4 group A member 3 (Nr4a3). The magenta module PPI had 25 nodes, 51 edges and a PPI enrichment *p*-value of <1.0 × 10^−16^ (Supplementary Figure S6).

There were 20 canonical pathways associated with the magenta module. The top canonical pathways of genes within the magenta module included CDK5 signaling, Il-6 signaling, PKR in interferon induction/antiviral response, corticotropic-releasing hormone signaling and CXCR4 signaling. There were three networks associated with genes within the magenta module, including: neurological disease, organismal injury and abnormalities, cell cycle (score of 27), dermatological diseases and conditions, organismal injury and abnormalities, organ morphology (score of 18) and cancer, gastrointestinal disease, and organismal injury and abnormalities (score of 8). The top diseases and functions associated with the magenta module were neurological disease, organism injury and abnormalities, gene expression, and cell cycle and cellular development.

### 2.4. DEGs Quantitative Real-Time PCR (qPCR) of Striatum and Olfactory Bulb

**Figure 6:**
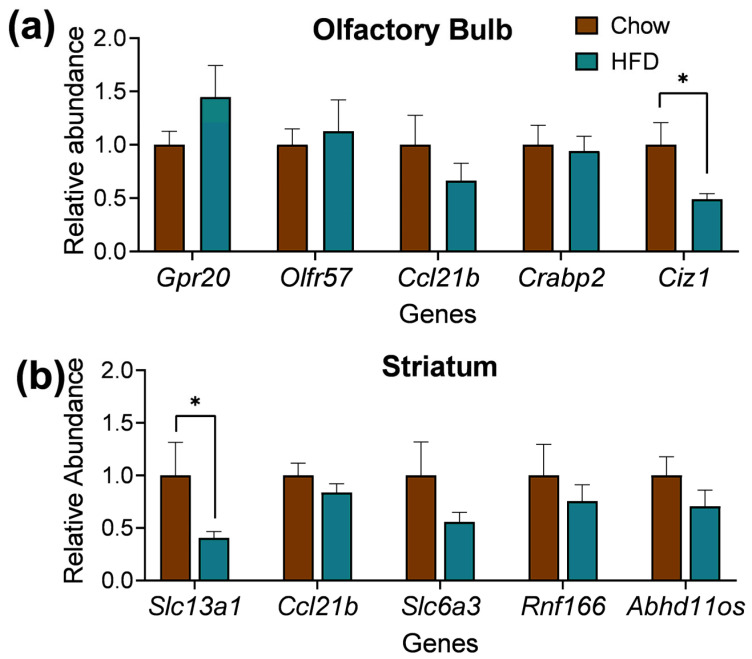
Gene expression analysis quantified by real-time PCR (qPCR). Five genes in each tissue were selected for RNAseq validation. Gene expression levels in the olfactory bulb (**a**); and striatum (**b**) were normalized to the housekeeping genes (Actb and Gapdh). Values are expressed in mean ± SEM and biological replicates varied from 9–13 for each diet. *Ciz1: t(24) = 2.385, *p* = 0.0253; *Slc13a1: t(11) = 2.720, *p* = 0.0199. * *p* < 0.05.

The findings from the qPCR experiments in the olfactory bulb (Figure 6a) and striatum (Figure 6b) served as a crucial validation for the accuracy and consistency of the RNAseq results. Most of the genes in both tissues followed the same tendency showed in the previously described RNAseq results. Changes in gene expression were measured in the striatum and olfactory bulb of the HFD group and regular chow group mice. Specifically, results from the RT-qPCR revealed a similar tendency of expression for the differentially expressed genes in both the olfactory bulb and the striatum, with the presence of a couple of non-concordant genes, consistent with previous findings [29]. Genes that were measured in the olfactory bulb included Gpr20, Olfr57, Ccl21b, Crabp2, and Ciz1. Genes that were measured in the striatal tissue included Slc13a1, Ccl21b, Slc6a3, Rnf166, and Abhd11os. These results strengthen our confidence in the identified gene expression changes within both regions, highlighting their biological significance.

### 2.5. Plasma Collection and Biomarkers Measurement

**Figure 7:**
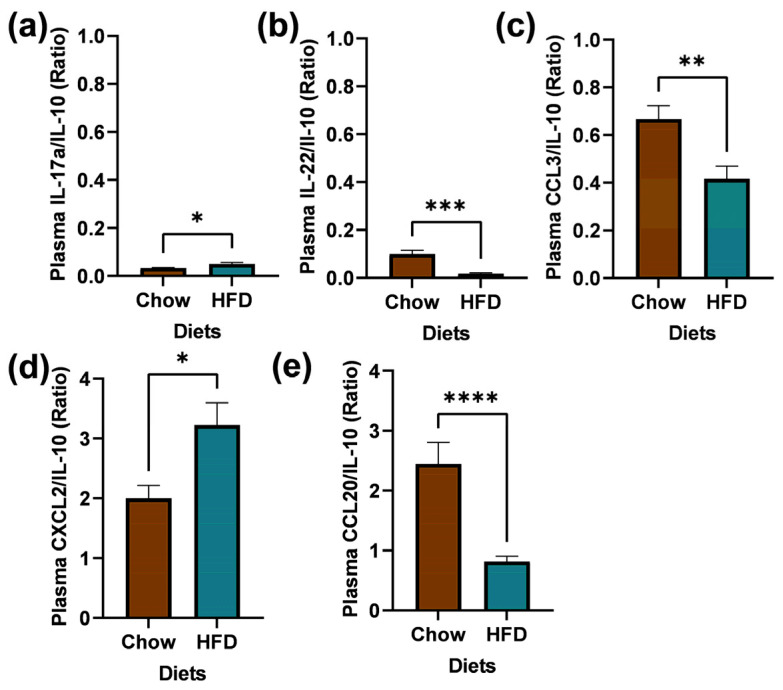
Plasma biomarkers. Ratio of pro- and anti-inflammatory of plasma biomarkers measured using the MSD. Values are expressed in ratio ± SEM, n = 4. (**a**) IL-17a/IL-10 U = 68, *p* = 0.0234; (**b**) IL-22/IL-10 U = 12, *p* = 0.0007; (**c**) CCL3/IL-10 U = 45, *p* = 0.0012; (**d**) CXCL2/IL-10 U = 61, *p* = 0.0108; (**e**) CCL20/IL-10 U = 22, *p* < 0.0001. * *p* < 0.05, ** *p* < 0.01, *** *p* < 0.001, **** *p* < 0.0001.

The biomarkers measurement findings are summarized in Figure 7. Plasma biomarker to IL-10 ratios IL-17a/IL-10 (U = 68, *p* = 0.0234), IL-22/IL-10 (U = 12, *p* = 0.0007), CCL3/UL-10 (10 U = 45, *p* = 0.0012), CXCL2/IL-10 (U = 61, *p* = 0.0108), and CCL20/IL-10 (U = 22, *p* < 0.001) were significantly different between the chow and HFD groups.

## 3. Discussion

Here, we identified DEGs in brain regions known to mediate feeding and metabolism in mice receiving different diets. We found 274 DE genes in the striatum and 11 in the olfactory bulb between obese HFD mice and normal-weight chow mice. In the striatum, we also identified 38 DEGs between animals fed a limited HFD compared to animals fed an ad libitum HFD and two DEGs between mice fed a limited HFD and those on the HFD-to-chow regimen. DEGs in both tissues included genes mediating inflammation (e.g., oxidative stress and inflammatory cytokine activation) and cell signaling (e.g., proliferation, migration, and apoptosis). DEGs in the striatum were also involved in mitochondrial dysfunction and reward pathways. These results suggest that diet can induce differential gene expression in the striatum and olfactory bulb and may contribute to inflammation, disruptions to cellular homeostasis and metabolism, and apoptosis. Lastly, when examining associations between WGCN and biological traits, we identified one module associated with free fatty acids. The hub genes in this module were associated with immune response, inflammation, and adipose tissue metabolism. However, the magnitude of the fold change ranged from small to moderate; while this may hold biologically significant implications, this should be considered when interpreting these results.

The pathogenesis of obesity is highly complex and involves multiple factors, including diet. Thus, we compared gene expression between animals receiving obesogenic and normal chow diets. In the striatum, the function of the top DEGs between the obese mice fed an ad libitum HFD and the normal-weight chow group suggests that the interaction between diet and obesity significantly impacts cell signaling (e.g., cell proliferation, decreased apoptosis, cell growth), immune response (e.g., autophagy), mitochondrial dysfunction, and inflammation (e.g., oxidative stress). The top five DEGs (Btg2, Rnf166, Abhd11os, Pcdh15, and Miat) are collectively involved in cell signaling, immune responses, and inflammation. Enrichment analyses of the 274 DEGs were consistent with the top five DEG functions, highlighting increased activity in pathways leading to apoptosis, inflammation, mitochondrial dysfunction, and other cell homeostasis alterations. DEG function coincides with the top functions of cancer and organism injury, and is characterized by disruptions to cell homeostasis, including increased cell proliferation, inhibition of apoptosis, and inflammatory signaling.

The DEGs between the ad libitum HFD and limited HFD groups are potentially indicative of changes specific to obesity. Like the ad libitum HFD group, the limited HFD group was exposed to the obesogenic nature of HFD; however, given the limited access to the diet, the mice did not gain excess weight. This group allowed us to explore the effects of HFD feeding in the absence of obesity. Thus, the DEGs between the HFD and the limited HFD groups allowed us to examine transcriptomic changes specific to obesity while controlling for exposure to the HFD. To the best of our knowledge, this is the first study to examine transcriptomic-level differences in striatal tissue using a dietary control group. The top five DEGs (Sp8, Btg2, Calb2, Asphd2, and Syne2) are involved in multiple cell/neuron signaling processes, including neuron generation, neuronal excitation, cell cycle progression. and cell migration and proliferation [11,15,16]. Functional enrichment analyses also suggest that there are differences in cell signaling and homeostasis between these two groups. Specifically, the ad libitum HFD group showed a downregulation of genes involved in integral cell signaling pathways and an upregulation of genes involved in cell signaling dysfunction, including changes in mitochondrial function, cell proliferation, and cellular migration. Functional enrichment analyses of the DEGs between the ad libitum HFD and limited HFD groups demonstrated that changes specific to obesity may be related to dysregulation of reward pathways, including dopamine signaling, obesity-induced cell signaling changes (e.g., cell cycle progression and cell migration), and neuronal activity (e.g., neuron generation and neuronal excitation) within the striatum. Notably, multiple reward-related genes are DEGs between the limited HFD and the ad libitum HFD groups. In addition to Sp8 (involved in the neurogenesis of D2 MSNs), other dopamine-related genes, including dopamine receptor 2 (Drd2) and adenosine receptor A (Adora2), are also DEGs. Drd2 expression was higher and Adora2 was lower in the HFD group than in the limited HFD group. Differences in Drd2 and Adora2 suggest that obesity (specifically, a decrease in ADORA2, which regulates proliferation and reduces apoptosis) causes dysregulation of dopamine signaling.

Additionally, obesity may be contributing to the dysregulation of reward processing by inducing dysfunction of neuronal activation. ADORA2 modulates neuronal excitability (e.g., the excitability of DRD2 neurons) and synaptic plasticity [30]. As mentioned above, CALB2 is also a modulator of neuronal excitability [16]. Thus, obesity may also be inducing dysregulation of neuronal excitability in reward centers (i.e., the striatum). Our findings indicating that obesity may contribute to the dysregulation of reward processing is consistent with transcriptomic changes in the striatum of postmortem brains of humans with obesity, in which the dysregulation of processes involved in dopamine-mediated reward saliency has been observed [31], and with human neuroimaging studies implicating altered or decreased dopaminergic receptor binding in the striatum [32,33,34] or brain regions that send efferent signals to the striatum [33]. Together, these consistent findings further support the continued use of mice as a strong model for the study of obesity-induced dopamine dysregulation [35,36,37,38,39].

A history of obesity may induce differential expression of genes involved in immunity and cell proliferation. A history of obesity may partially explain transcriptomic differences between the limited HFD and the HFD-to-chow groups. While the limited HFD group was never obese, the HFD-to-chow group represents obese mice that lost weight following a dietary intervention. Thus, the two DEGs between these two groups (Cd72 and EiF3jl) may be due to a recent history of obesity that had yet to resolve upon the normalization of body mass. The immune-related and cell signaling function of Cd74 and EiF3jl may be representative of residual obesity-related gene expression changes. The impact of a history of obesity on DEGs was also captured by the lack of DEGs between the HFD-to-chow and the ad libitum HFD groups, suggesting that gene expression changes induced by obesity may not fully recover following a dietary intervention. However, we did not identify any DEGs between the chow and HFD-to-chow groups, nor between the chow and limited HFD groups. The lack of DEGs suggests that, although the dietary intervention could not fully reverse the gene expression changes induced by obesity, the dietary intervention was sufficient to induce genomic changes that made the transcriptomic profile of the intervention group similar to that of the chow group. This lack of DEGs may indicate that there is some reversal of the genomic changes induced by DIO.

In the olfactory bulb, the identified DEGs suggest that the interaction between diet and obesity may impact lipids, the immune system, and inflammation. Changes in the olfactory bulb are striking because the olfactory function can impact energy balance regulation and obesity. The top five DEGs (Wfikkn2, Pcolce, Smoc2, Crabp2, and Ciz1) between the chow and ad libitum HFD groups were all upregulated and are involved in lipid deposition, immune response, and inflammation. The observed changes in lipid deposition are consistent with those in the literature reporting reduced fat deposits, insulin resistance, and increased thermogenesis in mice with ablated olfactory neurons [7]. The canonical pathways and their predicted activity are consistent with the function of the top five DEGs. The acute phase response signaling canonical pathway captures how weight gain contributes to the systemic inflammation underlying obesity and its comorbidities [40]. Similarly, gamma-glutamyl cycle proteins, including glutathione (GSH), play an essential role in metabolic dysfunction in obesity and other cardiometabolic pathological processes [41,42,43]. A recent study found that 14 days of HFD feeding altered GSH levels, likely via increased expression of IL-6 signaling in rat skeletal muscle [44]. The involvement of IL-6 is also consistent with the IPA molecular activity predictor in the HFD group of this study, which predicted the activation of the IL-6 signaling pathway.

Lastly, we found no DEGs between the limited HFD group and the chow group in either tissue. Both groups were not obese and maintained similar, normal weights throughout the study. The lack of DEGs between these groups suggests that dietary content alone (independent of obesity) did not significantly affect gene expression in the striatum or the olfactory bulb. In particular, the results suggest that there is not a chemical stimulus in the HFD per se that alters gene expression in at least the olfactory bulb.

Additionally, in the olfactory bulb, co-expressed genes within the magenta module were associated with free fatty acids and related to inflammation, immune response, and metabolism, suggesting that free fatty acid levels in the olfactory tissue are regulated by these pathways. Top hub genes (Egr3, Fosb, and Nr4a3) and top canonical pathways are involved in multiple biological pathways, including inflammation, immune response, and stress response (e.g., CRH signaling) [45,46,47,48]. The associated diseases and functions were consistent in highlighting the magenta module’s role in inflammation (e.g., IL-6 signaling and TNF signaling) and immune response (e.g., B lymphocytes and MIF regulation of innate immunity). Altogether, this suggests that the magenta module’s co-expressed genes may work together to mediate cellular dysfunction, including inflammatory responses and immune responses.

In summary, we found that DEGs in the striatum and olfactory bulb between HFD, chow, and limited-HFD-fed mice were associated with inflammation. Our results are consistent with a large number of studies in the literature indicating that obesity is characterized by systemic chronic inflammation [49,50,51,52,53]. Inflammation is also related to mitochondrial dysfunction and other energy disturbances leading to oxidative damage in the cell [49]. Specifically, our findings utilized transcriptomic analyses and WGCNA to build upon studies reporting diet-induced mitochondrial changes and neuroinflammation of the cerebral cortex, hypothalamus, cerebellum, and amygdala in rodents [49,50,51,52], by showing that neuroinflammation is also found in striatal and olfactory tissue, even after dietary interventions.

There are limitations to this study. While we reported on and examined DEGs that were highly statistically significant even after adjusting for multiple comparisons, the magnitude of change ranged from small to moderate. These genes were included in our study, as biologically relevant functions occur even at small fold changes in RNA levels [54]. Although even smaller fold changes may be meaningful and biologically significant, larger changes might be required to draw strong conclusions and should be considered when interpreting the results. Future studies should examine these gene functions using knockout models specific to these tissues or utilize pharmacological antagonists and agonist to determine the role of the genes and their products in phenotypic and behavioral responses to the various diets. In addition, live imaging of neuronal activity within these regions, through photometry and other methods, could help to validate the contribution of specific DEGs to dietary-induced neuronal activity changes. In addition, a longer duration of dietary manipulations could help to determine if increased diet longevity enhances the observed results or captures other changes that we were unable to observe during this study’s 12-week duration. Furthermore, transcriptomic-level differences across diets should also be examined in larger animal cohorts and across sexes and age groups. We utilized whole striatum and olfactory bulb; future studies could examine single-cell RNA-sequencing to determine whether DEGs are cell-type specific. Lastly, obesity has a multifactorial etiology, and its complex pathogenesis cannot entirely be explained by dietary differences alone [2,55]. Genetic predisposition, environmental exposures, and social factors, also play a role in the pathogenesis of obesity [2,55]. The results and interpretation of the effect of the genes discussed in this study should also be considered in the context of the multidimensionality of obesity.

This study’s strengths include examining transcriptomic-level differences across two tissues within animals challenged with multiple diets. Additionally, few studies have examined such differences by controlling for dietary content and dietary interventions. We also utilized WGCNA to examine if groups of co-expressed genes were associated with any biological or behavioral traits. Few studies have examined system-level changes and their correlation to both biological and activity measures. Additionally, few studies have explored transcriptomic-level differences associated with diet in striatum and olfactory tissue.

## 4. Materials and Methods

### 4.1. Animal Handling and Diets

One cohort of forty male 18-weeks old C57BL/6 mice (10 mice from 4 litters, all litters were equally divided among the four experimental groups) were housed under a 12:12 light–dark cycle and received ad libitum access to food and water unless otherwise stated. Mice were randomized into four groups (Figure 1a) receiving either a conventional normal chow diet (n = 10), an ad libitum HFD (60% kcal%/fat; Research Diets, diet D12492; n = 10) or limited HFD (n = 10) for 12–13 weeks. Additionally, a fourth group of animals received six weeks of an HFD (60% kcal%/fat; Research Diets, diet D12492) followed by six weeks of a normal chow diet (n = 10), which we refer to as the HFD-to-chow group. The limited HFD group received a normocaloric amount of HFD based on the chow group’s average daily caloric intake and thus was, in effect, a pair-feeding control. We calculated, weighed, and administered the amount of HFD with an equivalent caloric value. The body weights of the four groups are depicted in Figure 1b. All procedures involving animals were reviewed and approved by the National Institute of Diabetes and Digestive and Kidney Diseases Animal Care and Use Committee (IACUC; #K010-DEOB-16). Animals were treated, and the studies were conducted, in accordance with the National Institutes of Health Animal Welfare guidelines. The study was carried out in compliance with the ARRIVE guidelines.

### 4.2. Sample Collection

Mice were killed by CO_2_ and exsanguinated. Trunk blood was collected to measure biological traits. Immediately following exsanguination, the whole striatum and olfactory bulb were removed and flash-frozen in liquid nitrogen for RNA extraction and sequencing. Extracted brain regions are depicted in Figure 1c.

### 4.3. Biological Measurements

Trunk blood was centrifuged (10,000 rpm for 6 min) to collect serum and stored at −80 °C until assayed. An enzyme-linked immunoabsorbent assay (ELISA) kit was used to measure leptin (ng/mL) (R&D Systems, Minneapolis, Minnesota) [56]. Radioimmunoassay (RIA was used to measure insulin (Millipore, St. Charles, MO, USA). Indirect calorimetric assays were used to measure free fatty acids (Roche Diagnostics Gmbh, Mannheim, Germany), cholesterol (Thermo Scientific, Middletown, VA, USA), and triglycerides (Pointe Scientific Inc., Canton, MI, USA). Glucose was measured using a glucometer (Bayer Corp., Elkhart, IN, USA). Body weight was measured weekly; changes in body weight were calculated using these values.

### 4.4. Activity Measurements

Activity was quantified as described by Matikainen-Ankney et al. [57]. Briefly, physical activity levels were measured using custom-built activity sensors (PMID: 32133782) placed in the home cage for the experiment’s duration. Logged data were analyzed using custom Python scripts (available at https://osf.io/kw4v6/ accessed on 20 December 2023).

### 4.5. RNA Sequencing

Total RNA was extracted using Trizol reagent (Thermo Fisher/Invitrogen, Waltham, MA, USA), micro-tissue homogenizers (Biomasher II), and RNeasy Mini Kits (Qiagen, Valencia, CA, USA). The integrity of the total RNA was assessed using an Agilent 2100 BioAnalyzer (Agilent, Santa Clara, CA, USA). The RNA integrity number [11] of all samples was greater than 9. RNA samples were treated with Ribo-Zero Gold rRNA Removal Kit (Illumina, San Diego, CA, USA), and a library for RNA sequencing was prepared using TruSeq Stranded Total RNA Kit (Illumina). The library was paired and sequenced on HiSeq 2500 system (Illumina) with 100 sequencing cycles. Investigators were blinded during RNA extraction and sequencing, except during data analysis, where animals were grouped together to enable comparisons.

### 4.6. Analysis of RNA Sequencing

DE analysis and gene count values were entered into DESeq2 R-4.4.1 software https://cran.r-project.org/bin/windows/base/. The gene count values were combined from individual samples, so they were not normalized across tissues or subjects. The DESeq2 software internally normalized gene count values (using GLM and Bayes approaches) and generated *p*-values of DEGs based on its internal processing. *p*-values for DEGs were corrected for false discovery rates (FDR) [58]. The RNA-sequencing workflow is summarized in Supplementary Figure S1; the principal component analyses (PCA) plot is depicted in Supplementary Figure S2.

### 4.7. Enrichment Analysis

Functional enrichment analysis was performed using the online version of GOStat (http://gostat.wehi.edu.au/cgi-bin/goStat.pl accessed on 18 January 2021) [59]. Parameters were chosen as follows: database: mgi; minimal length of considered GO paths: 3; maximum number of GOs: 30; cluster GOs: 5; direction: over-represented; correct for multiple testing: false discovery rate. Maximal *p*-value parameters were set to 0.05 for the 274 DEGs in the striatum. The maximal *p*-value was adjusted to 0.1 for the 38 DEGs (between the limited HFD and the HFD) and the 11 DEG in the olfactory bulb to capture overall gene associations in this smaller number of DEG. All genes detected through our RNA-sequencing analysis (17,542 HFD vs. chow in the striatum; 17,568 limited HFD vs. HFD in the striatum; 17,453 HFD vs. chow in the olfactory bulb) were used as a genomic background to examine the enrichment of DEGs.

### 4.8. Ingenuity Pathway Analysis (IPA)

IPA.Ika © 2024 QIAGEN Digital Insights (https://www.qiagenbioinformatics.com/products/ingenuity-pathway-analysis/ accessed on 20 August 2024) was used for the functional analysis. DEGs were inputted into IPA to identify canonical pathways and networks associated with these genes. Canonical pathways identified key biological pathways, and molecular activity predictor analyses of these canonical pathways were conducted to predict the neighboring molecules’ activity. Networks were constructed to identify and visualize gene–gene interactions, with each connection representing known relationships between genes. Identified networks were scored based on the degree of relevance (i.e., -log (Fisher’s Exact test)) of their genes with a list of biological functions stored in the Ingenuity Knowledge Base. Functions and diseases that overlapped/included our DEGs were also identified using IPA.

### 4.9. Construction of Weighted Gene Co-Expression Networks

The WGCNA R software package (version 1.72-5) was used to construct co-expression networks. One-step network construction and module detection were conducted using the following parameters: TOMType  =  ”unsigned”, minModuleSize  =  10, reassign Threshold  =  0, and mergeCutHeight  =  0.25. Subsequently, an eigenvector (vector associated with linear system equations) was computed for each detected gene module. Then, associations between these modules and biological measurements (e.g., free fatty acid levels, leptin levels and change in body weight) and activity measures were assessed based on Pearson’s correlation between each module’s eigenvector and each trait and activity measure. *p*-values for module-trait associations were corrected for false discovery rate (FDR) using the Benjamini–Hochberg procedure [58].

### 4.10. Identification of Hub Genes

Hub genes, genes with high connectivity, were identified using topology similarity analysis, as described by Joseph et al. [60]. In brief, to determine within-module connectivity, a 99% topology overlap matrix was computed and utilized as a cutoff to determine gene connectivity. Higher topological overlap suggests the two genes are more likely to belong to the same functional class [56,61]. High connectivity was defined as geneModuleMembership > 0.80 and a geneTraitSignificance > 0.20. The genes identified as hub genes were input into the search retrieval of interacting genes (STRING) to construct and visualize protein–protein interactions (PPIs). The top 10% of genes with the highest connectivity were classified as top genes.

### 4.11. Quantitative Real-Time PCR (qPCR) of Striatum and Olfactory Bulb

Brains from two cohorts of chow and HFD C57BL/6 male mice (n = 20, PND 126) were flash-frozen and kept at −80 °C until further processing. Then, snap-frozen brains were transferred to a Leica CM3050S Cryostat until acclimated, and 50 µm longitudinal cryosections were cut. One striatum and olfactory bulb punch per animal were collected, and total RNA was extracted with RNeasy Plus Micro Kit following the manufacturer’s instructions. Total RNA quality/quantity was analyzed using the RNA 6000 Pico kit on an Agilent 2100 Bioanalyzer (Agilent, Santa Clara, CA, USA). Samples with an RNA integrity number (RIN) >7 were immediately used for complementary DNA (cDNA) synthesis. Total RNA (7.6 ng) was reverse-transcribed using the Advantage RT-for-PCR Kit (Takara, San Jose, CA, USA), as per the manufacturer’s instructions. Prior to use, the cDNA was diluted 1:5 with RNase/DNase free water.

Quantitative RT-PCR (qPCR) was performed in triplicate in 10 µL using the QuantStudio™ 6 Flex Real-Time PCR System in a 384-well plate. Each reaction contained TaqMan Universal PCR Master Mix, 20x TaqMan Gene Expression Assay, RNase-free water, and diluted cDNA according to the manufacturer’s protocol. In each reaction, 2 µL of the diluted cDNA was used. The TaqMan Gene Expression Assays used were mBtg2 (Mm00476162_m) Rnf166 (Mm01242213_m1), Abhd11os (Mm01201751_g1), Pcdh15 (Mm00480870_m1), Miat (Mm01196418_g1), Smoc2 (Mm00491553_m1), Ciz1 (Mm00503766_m1), Pcolce (Mm00476608_m1), Wfikkn2 (Mm00725281_m1), Crabp2 (Mm00801693_g1), Sp8 (Mm01701281_m1), Btg2 (Mm00476162_m1), Calb2 (Mm00801461_m1), Asphd2 (Mm01201942_m1), Syne2 (Mm00621101_m1), Gpr20 (Mm02620726_s1), Slc13a1 (Mm00490339_m1), Slc6a3 (Mm00438388_m1), Olf57 (Mm00730842_s1), Trpm1 (Mm00450619_m1), Ccl21a, Ccl21b, Gm (Mm03646971_gH), Adcy10 (Mm00557236_m1). Actin (Actb) and glyceraldehyde-3-phosphate dehydrogenase (Gapdh) were used as reference genes for every sample. Additionally, each run contained samples with the reverse transcriptase omitted. The mixture was initially activated by holding at 50 °C for 2 min and then heating at 95 °C for 10 min, followed by 40 cycles of denaturation steps at 95 °C for 15 s and annealing/elongation at 60 °C for 1 min.

qPCR data was analyzed using the baseline set by the manufacturer. A two-tailed unpaired t test with a 95% confidence level was used to evaluate transcript level differences in GraphPad Prism 9.

### 4.12. Plasma Collection and Biomarker Measurement

Trunk blood samples from one cohort of chow and HFD C57BL/6 male mice (n = 4, PND 126) were collected. Plasma was separated using microvette CB 300 tubes (Sarstedt AG & Co, Numbrecht, Germany) and centrifuged at 1500x *g* for 15 min. Then, plasma samples were diluted 2-fold in sample diluent containing cOmplete Mini EDTA-free protease inhibitor (Roche, IN, USA). Plasma was tested in duplicate in the U-plex biomarker 29-Plex as per the manufacturer’s protocol (K15355K, Meso Scale Discovery, Rockville, MD, USA). The electrochemiluminescence signal for each cytokine and chemokine was measured on a MESO QuickPlex SQ 120 instrument (Meso Scale Diagnostics, Rockville, MD, USA). The analysis was conducted using the DISCOVERY WORKBENCH version 4.0 software and GraphPad Prism 9.

## 5. Conclusions

Overall, our results suggest that the interactions of diet and obesity are sufficient to induce transcriptomic-level changes to inflammatory and immune-related genes in the striatum and the olfactory bulb. Additionally, DIO led to mitochondrial dysfunction and dopamine-related dysregulation in striatal tissue. Future studies should continue to explore nutrition-related transcriptomic changes and their underlying mechanisms.

## Figures and Tables

**Table 1:** Top 10 differentially expressed genes in the striatum.

Striatum: HFD vs. Chow		
Gene	Logarithm of Fold Change	FDR-Adjusted *p*-Value
*Btg2*	0.8318072	8.66 × 10^−6^
*Rnf166*	−0.4600948	1.32 × 10^−5^
*Abhd11os*	−0.8731612	0.00010783
*Pcdh15*	0.9363582	0.000422444
*Miat*	0.4841786	0.000422444
*Gng10*	−0.3782426	0.000485772
*Tesc*	−0.5258876	0.000551012
*Col12a1*	1.3235917	0.000638062
*Hmgn2*	−0.4035872	0.001924701
*Dcx*	0.4088739	0.002103913
Striatum: Limited HFD vs. HFD		
*Sp8*	−1.105220599	0.000614072
*Btg2*	−0.920027322	0.003250265
*Calb2*	−1.087379338	0.00490404
*Asphd2*	0.387021075	0.010988066
*Flywch2*	1.035088914	0.011978881
*Gng12*	−0.274927851	0.011978881
*Scgn*	−2.114478585	0.011978881
*Syne2*	−0.414699518	0.011978881
*Adamts1*	−0.885121012	0.019887626
*Hmgn5*	−0.31248415	0.019887626

**Table 2:** Top 10 differentially expressed genes in the olfactory bulb.

Striatum: HFD vs. Chow		
Gene	Logarithm of Fold Change	FDR-Adjusted *p*-Value
*Smoc2*	0.8872776	0.02427586
*Ciz1*	0.3847122	0.02427586
*Pcolce*	1.1616673	0.02427586
*Wfikkn2*	1.3674595	0.02427586
*Crabp2*	0.7584510	0.02427586
*Fn1*	0.5652448	0.02427586
*Ndrg1*	0.2978863	0.03900604
*Anpep*	1.0962185	0.03900604
*1500015A07Rik*	−0.4177287	0.04459341
*Steap3*	0.5881312	0.04674888

## Data Availability

The data presented in this study are available on request from the corresponding author due to ongoing study.

## Supplementary Materials

The following supporting information can be downloaded at: https://www.mdpi.com/article/10.3390/ijms25179330/s1.

## Abbreviations

DE: differentially expressed; FDR: False discovery rate; HFD: high fat diet; IPA: Ingenuity Pathway Analysis; WGCNA: weighted gene correlation network analysis.
