# Supplementary material for: Diet-Induced Obesity Induces Transcriptomic Changes in Neuroimmunometabolic-Related Genes in the Striatum and Olfactory Bulb
Source: Int J Mol Sci. 2024 Aug 28;25(17):9330. doi: 10.3390/ijms25179330 (PMC11395036; doi:10.3390/ijms25179330)
Supplement: Supplementary file 1 [file ijms-25-09330-s001.zip › ijms-3133022-supplementary.pdf]

SUPPLEMENTARY TABLES

Table S1. Enriched GO terms in the clinical trait-associated modules

	**Enriched GO terms**	**Count**	**Total**	**p-value**	**FDR-adjusted *p*-value**
Striatum					
**HFD vs chow**	GO:0050136: (M) NADH dehydrogenase (quinone) activity	8	35	1.57e-0	7.85e-05
	GO:0008137: (M) NADH dehydrogenase (ubiquinone) activity	8	35	1.57e-07	7.85e-05
	GO:0003954: (M) NADH dehydrogenase activity	8	35	1.57e-07	7.85e-05
	GO:001665: (M) oxidoreductase activity	8	37	2.49e-07	9.36e-05
	GO:001661: (M) oxidoreductase activity, acting on NAD(P) H	8	52	3.84e-06	0.00116
	GO:0009055: (M) electron transfer activity	9	108	0.000147	0.0324
	GO:0005747: (C) mitochondrial respiratory complex 1	3	7	0.000194	0.0324
	GO:0030964: (C) NADH dehydrogenase complex	3	7	0.000194	0.0324
	GO:0045271: (C) respiratory chain complex 1	3	7	0.000194	0.0324
**limited HFD vs HFD**	GO:0042221: (P) response to chemicals	4	253	0.00216	0.117
	GO:0032230: (P) regulation of low-density lipoprotein receptor catabolic process	1	1	0.00221	0.117
	GO:0032799: (P) low-density lipoprotein receptor particle metabolic process	1	1	0.00221	0.117
	GO:0032803: (P) regulation of low-density lipoprotein receptor catabolic process	1	1	0.00221	0.117
	GO:0032801: (P) receptor catabolic process	1	1	0.00221	0.117
	GO:0032802: (P) low-density lipoprotein particle receptor catabolic process	1	1	0.00221	0.117
	GO:0001611: (F) G protein-coupled adenosine receptor activity	1	1	0.00221	0.117
Olfactory bulb					
**HFD vs chow**	GO:0001972: (F) retinoic acid binding	1	1	0.000819	0.0848
	GO:0030332: (F) cyclin binding	1	2	0.00164	0.0848
	GO:0000293: (F) ferric-chelate reductase activity	1	4	0.00327	0.0848
	GO:0007044: (P) cell-substrate junction assembly	1	4	0.00327	0.0848
	GO:0042573: (P) retinoic acid metabolic process	1	5	0.00409	0.0848
Letters represent gene ontology (GO) domains where: F: molecular function; C: cellular component; P: biological process.					

SUPPLEMENTARY FIGURES

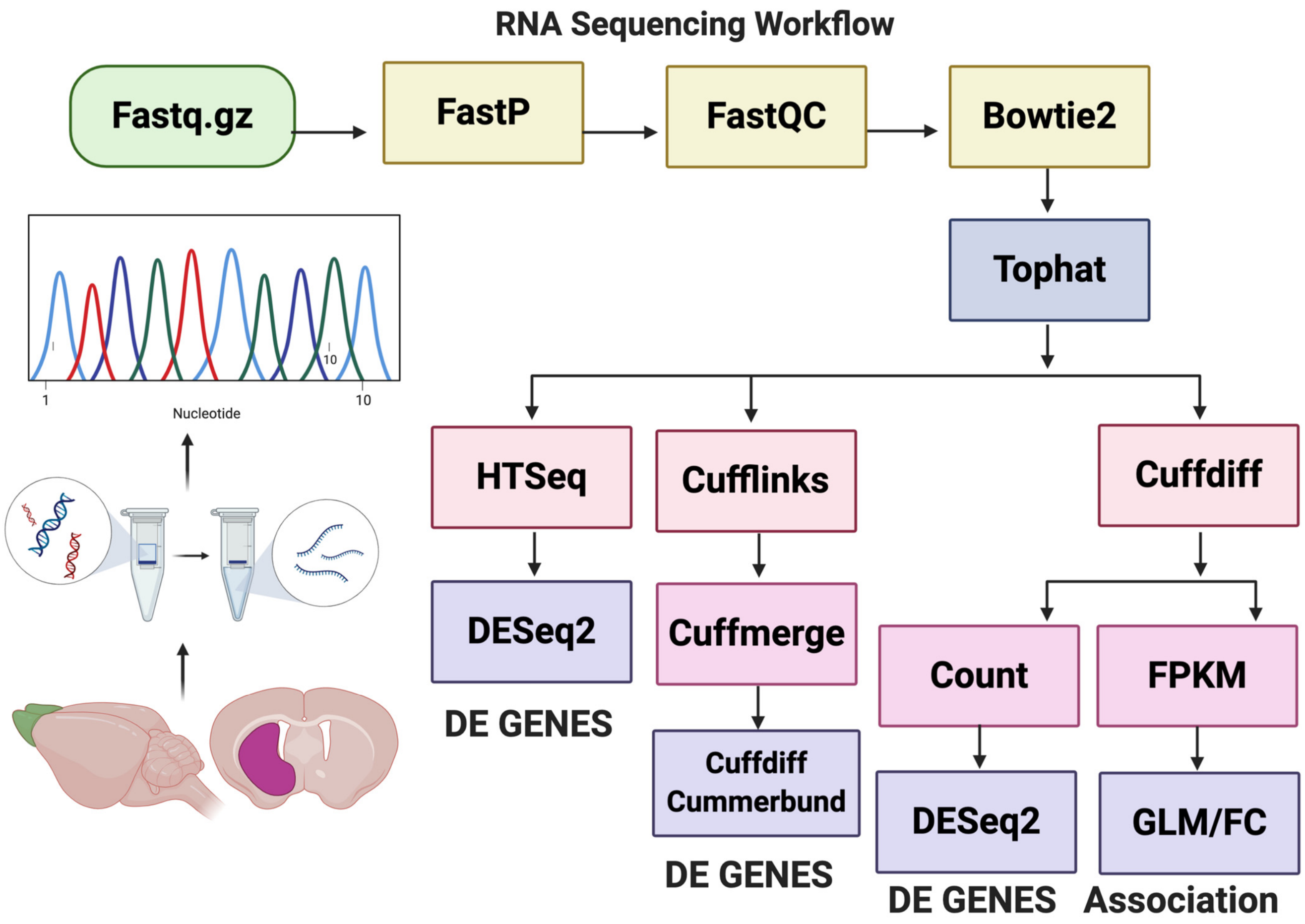

Figure S1. RNA Sequencing workflow.

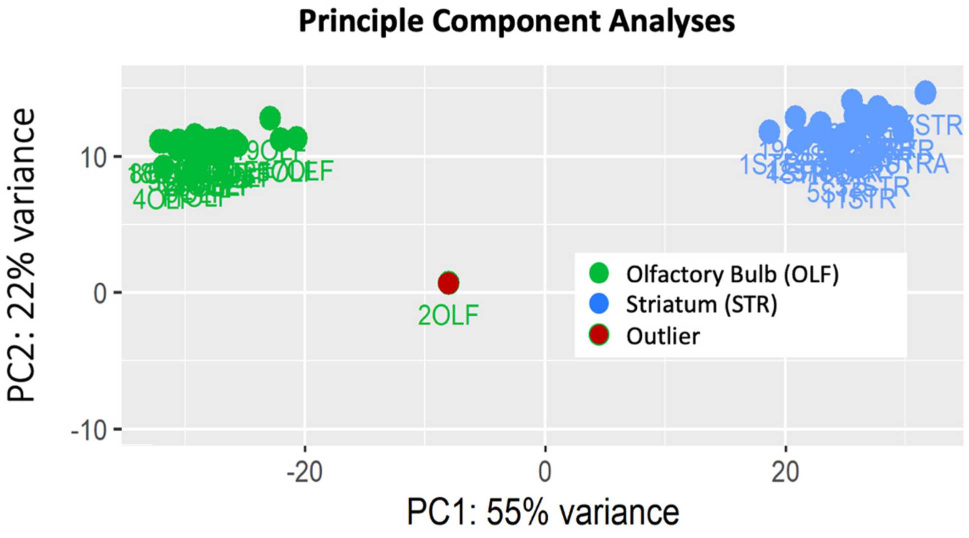

Figure S2. Principle Component Analyses (PCA). PCA plot displaying all 48 samples (24 olfactory bulb and 24 striatum). PCA shows expected grouping among tissue sample groups across the two PCs. PC1 accounts for 55% of variance and PC2 accounts for 22% of the variance. PC analysis was applied to normalized (reads per kilobases of transcript per 1 million mapped reads) and log-transformed count data. One outlier (2OLF) was identified and removed from all analyses. STR: striatum, OLF: olfactory bulb

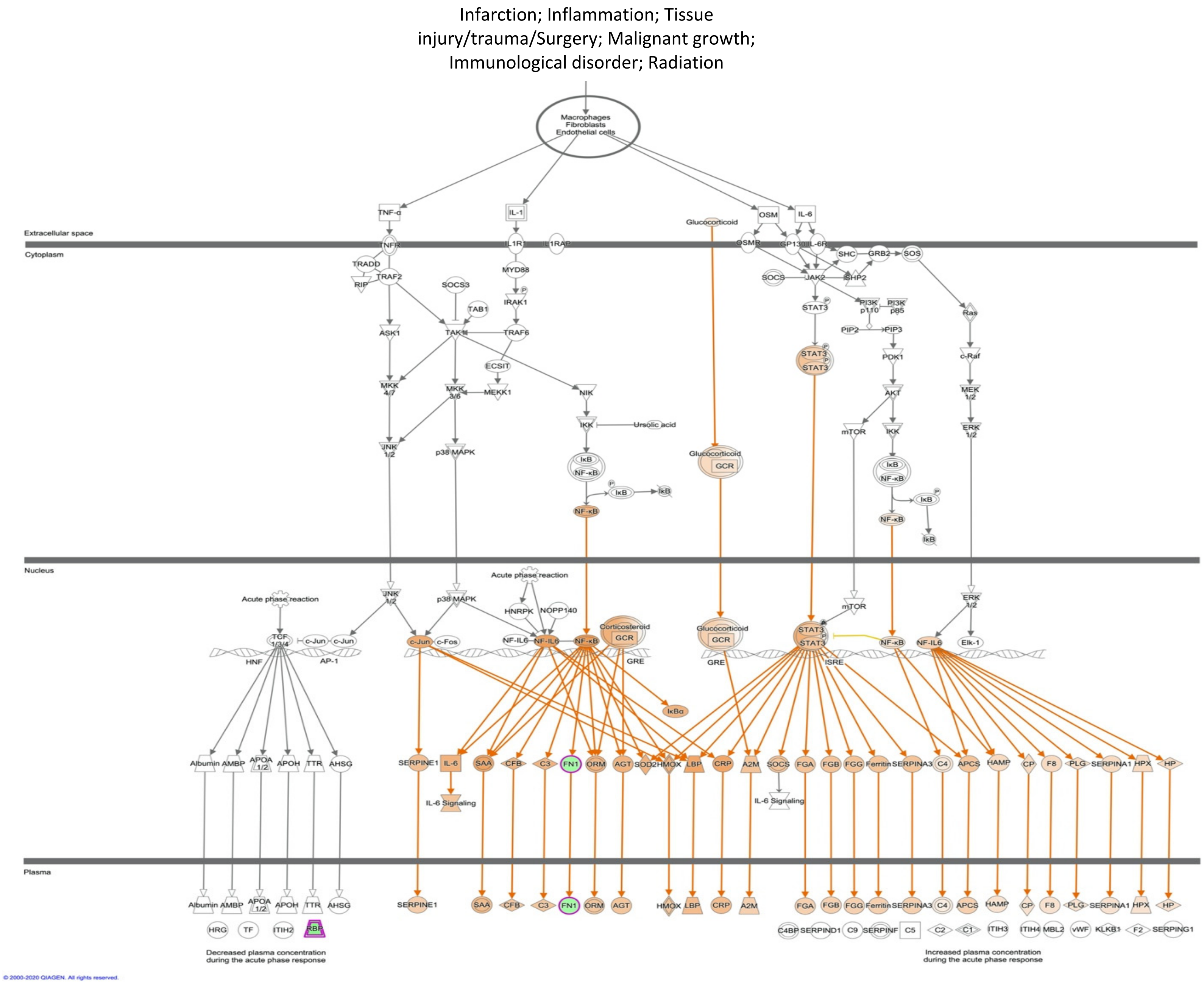

Figure S3. Acute Phase Response Signaling Canonical Pathway. Genes in green are upregulated genes. Colored arrows show the results of the Molecule Activity Predictor; orange lines represent pathway activation; blue lines represent inhibition; yellow lines represent conflicting data.

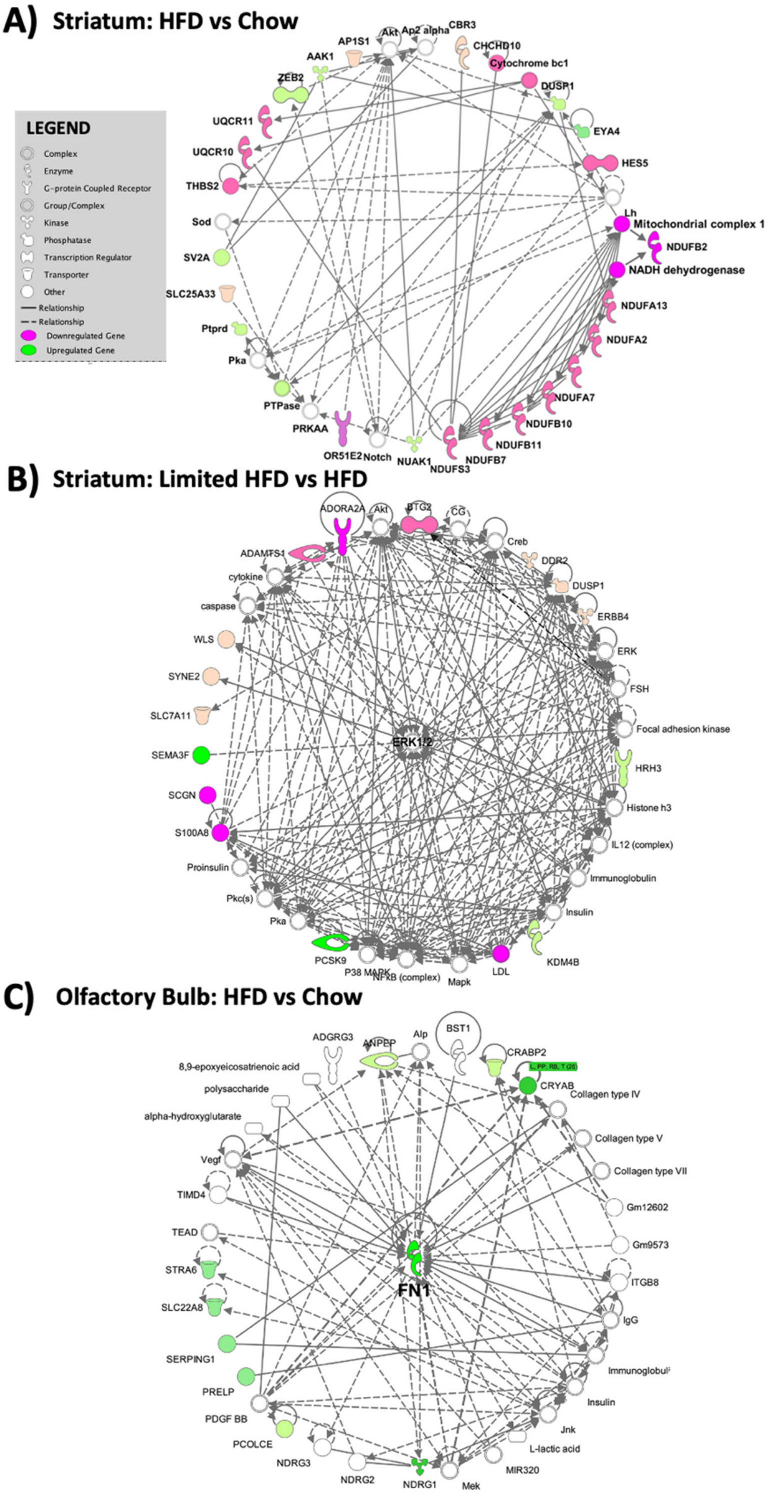

Figure S4. Top Network of Differentially Expressed Genes. A) Top network of DE genes between HFD and chow groups in the striatum. B) Top network of DE genes between the limited HFD and HFD groups. C) Top network of DE genes between the HFD and chow groups in the olfactory bulb. Genes colored green are significantly ($p<0.05$) upregulated and magenta are downregulated. Darker colors correspond to lower p-values. Figure was generated through the use of IPA (QIAGEN Inc., https://www.qiagenbio-informatics.com/products/ingenuity-pathway-analysis)

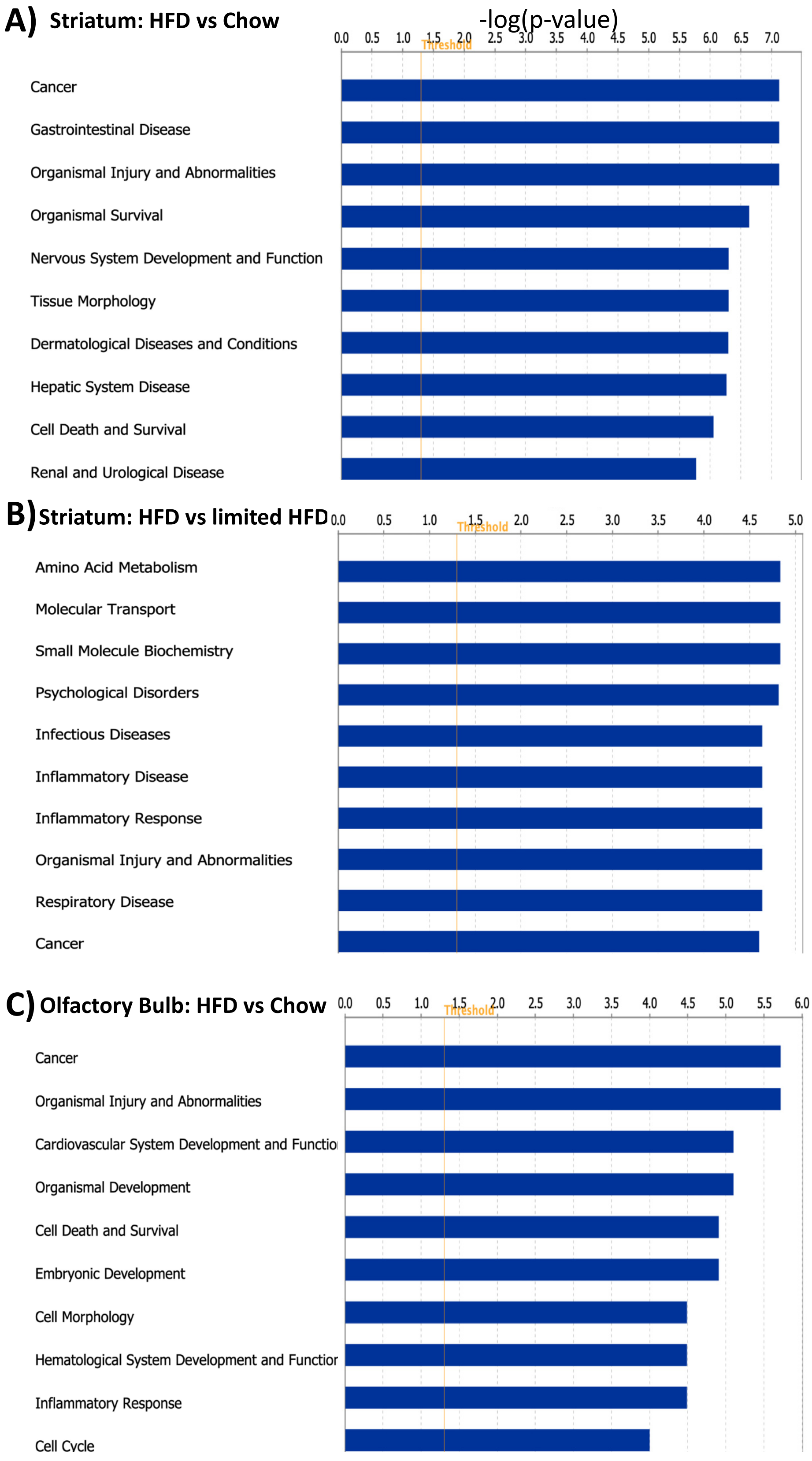

Figure S5. Diseases and Biological Function of Differentially Expressed Genes
This figure was generated through the use of IPA (QIAGEN Inc., https://www.qiagenbioinformatics.com/products/ingenuity-pathway-analysis).

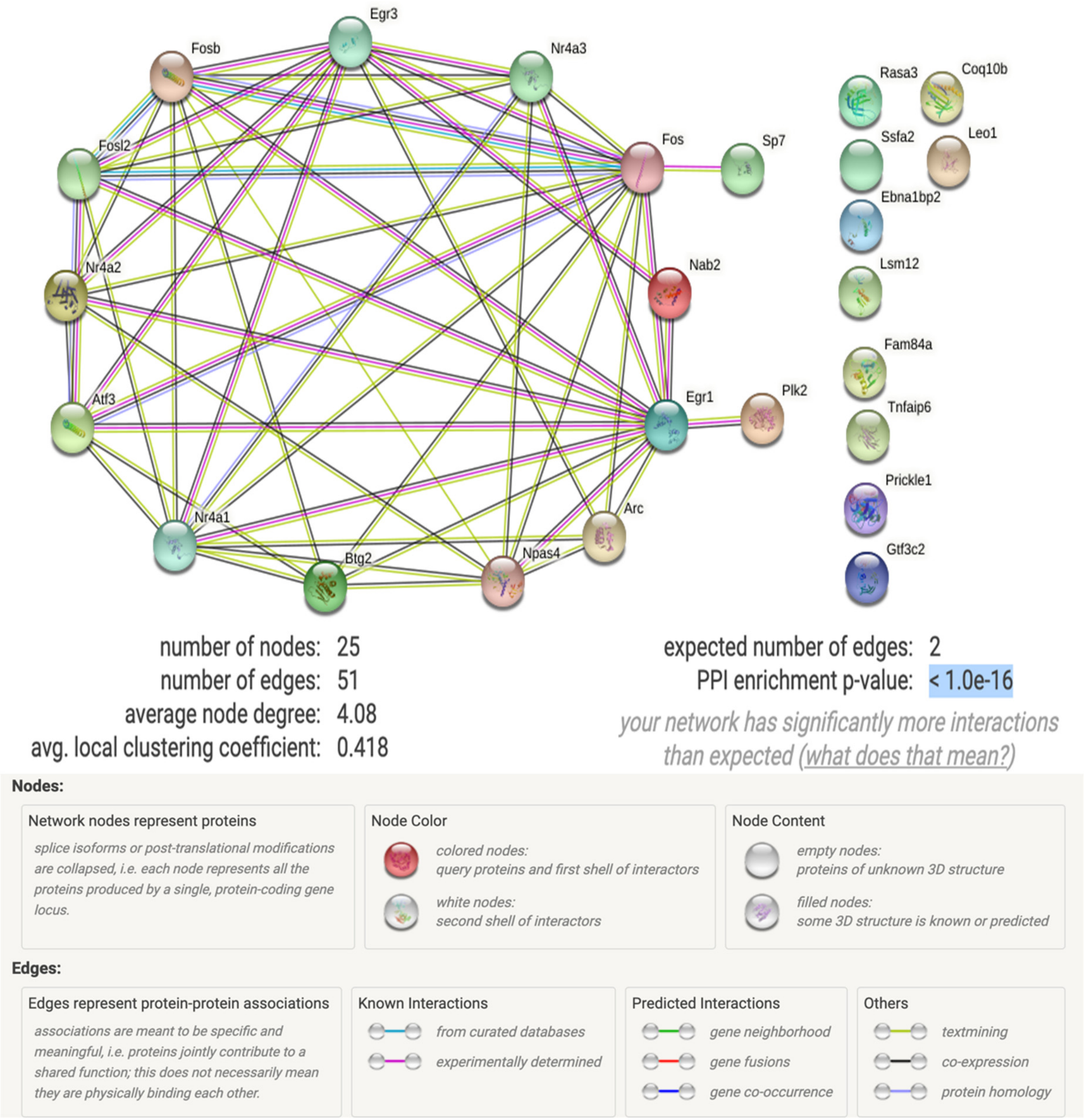

Figure S6. Protein-protein Interactions Depicting Hub Genes. Protein-protein interaction between the hub genes, Egr3, Nr4a3, Fosb, Fos, Nab2, Nr4a2, Egr1, Fam84a, Fosl2, Npas4, and Arc.

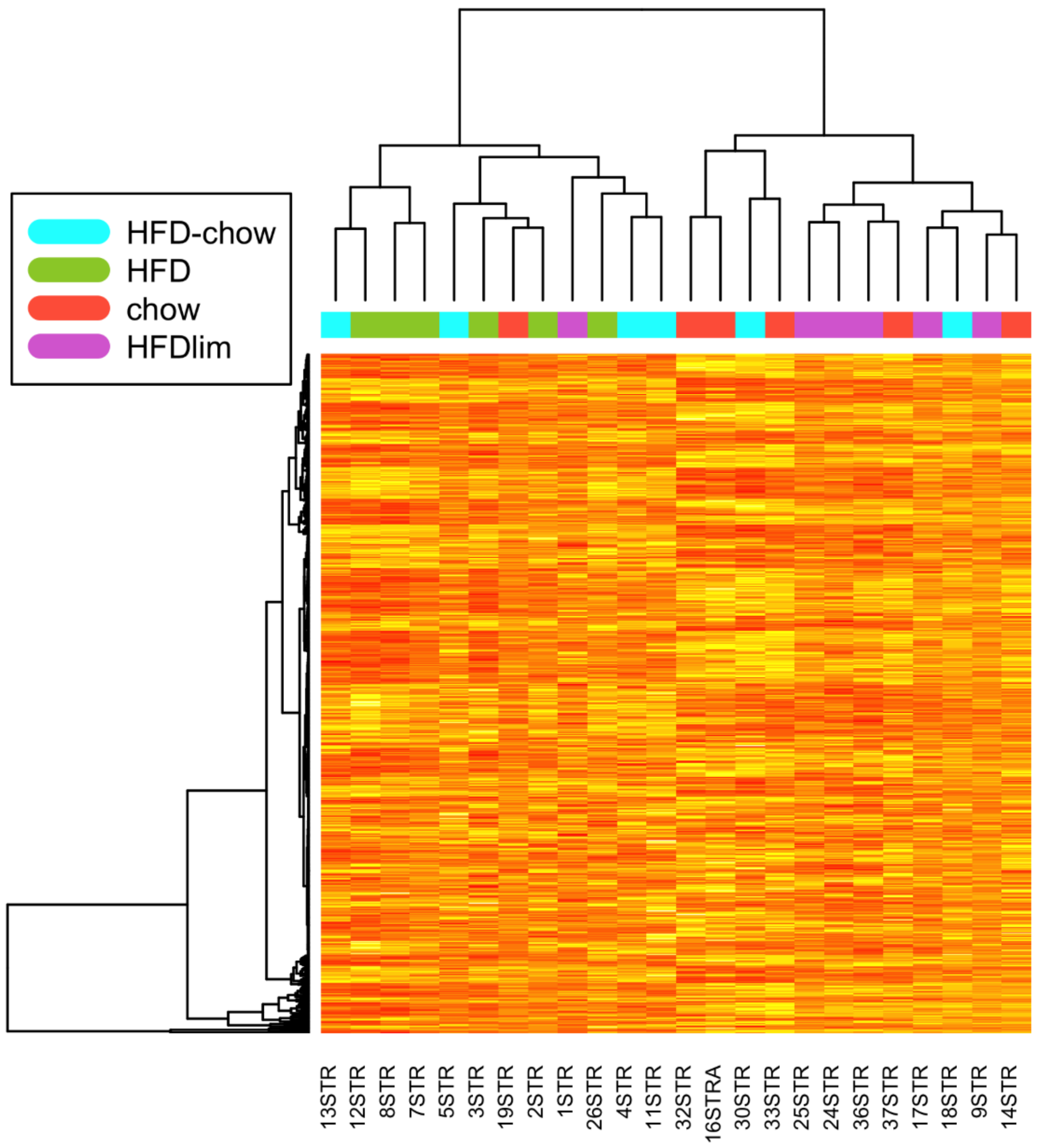

Figure S7. Group similarity analysis by diet in STRIATUM
Numbers represent STR samples across groups.
